# 

**Published:** 1913-12-13

**Authors:** 


### CONTENTS.

| | PAGE |
|---|---|
| **Hospital Consultants and Panel Practice** ... | 271 |
| **Combination of Mental Hospital Staffs** ... | 272 |
| **Hospital and Institutional News,** including— ... | 273 |

THE HOSPITAL'S Christmas Supplement—Sir Henry Burdett's Reports—The British Hospitals' Association Meeting, 1914—The London Hospital—A Poser for the Deans—Infirmary Chairman Assaulted—Venereal Diseases: an Australian Experiment—The Fate of the Ambulance Scheme—A Cottage Hospital Closed—School Medical Officers at Loggerheads—The Sight of the Blind—Telephones at Nursing Homes—Departmental Changes at the London—The End of the Dimock Case—The Issue of False Death Certificates—Insurance Act and Infirmary Patients—Doctor and Bursar in One—The Orphanage in Institutional Life—The Proposed Amalgamation of St. Thomas's and Westminster Hospitals—St. George's Hospital—The Grave Danger of St. George's Hospital—The Latest Use for *X*-Rays—The Conditions of Service at London Mental Hospitals—The Hospital and Infirmary Doll Show—A Successful Linen League—A Phenomenal Hospital Bazaar—Doctor's Death from Septicæmia—This Week's Drug Market.

| | PAGE |
|---|---|
| **The Modern Orthopædic Unit** ... ... ... | 279 |
| IV. The Out-patient Division (illustrated). | |
| **A Central Public Laboratory Clinic in Hospital and Private Practice** ... ... ... ... | 281 |
| By DAVID WALSH, M.D. | |
| **Report on the Great Yarmouth Hospital** ... | 283 |
| By Sir HENRY BURDETT, K.C.B., K.C.V.O. | |
| **Differing Systems of Patients' Contributions** | 285 |
| **Discontent in the Mental Hospital Service** ... | 286 |
| Combination Among Assistant Medical Officers. | |
| **The Statistical Report of King Edward's Fund** (continued) ... ... ... ... ... ... ... | 287 |

### CONTENTS—(*continued*).

| | PAGE |
|---|---|
| **Tuberculosis Dispensaries in Theory and Practice** ... ... ... .. ... ... | 289 |
| By F. J. C. BLACKMORE, M.R.C.S., L.R.C.P. | |
| **Origin and Evolution of the 18th Century Hospital Movement** ... ... ... ... | 290 |
| **National Insurance Act Developments** ... ... | 291 |
| Some Likely Effects of Excessive Sickness Claims. | |
| **Hospital Architecture and Construction** ... | 293 |
| The Children's Hospital, Sunderland, illustrated with plans. | |
| **The Harley Institute** ... ... ... ... ... | 294 |
| **Fire at Winsley Sanatorium** ... ... ... | 294 |
| **The Editor's Letter-Box** ... ... ... ... ... | 295 |
| St. George's Hospital. Confidential Reports and their Abuses. | |
| **The Profession of Medicine** ... ... ... | 296 |
| Panel or Anti-Panel? Why Practitioners must make their Choice. The Panel and the Politician. | |
| **The Institutional Worker** ... ... (Suppl.) | 1 |
| Workpeople's Hospital Funds in Small Industrial Centres. Questions for December. | |
| **Our Bureau of Information** ... ... „ | 2 |
| Boiler Insulating Material. The Sick and in Need. Employment and Training. Miscellaneous Information. | |
| **Buildings Contemplated and in Progress** „ | 3 |
| **The Book Market** ... ... ... „ | 3 |

